# Underwater endoscopic mucosal resection for colorectal lesions: Can it be an “Underwater” revolution?

**DOI:** 10.1002/deo2.84

**Published:** 2022-01-09

**Authors:** Yoji Takeuchi, Satoki Shichijo, Noriya Uedo, Ryu Ishihara

**Affiliations:** ^1^ Department of Gastrointestinal Oncology Osaka International Cancer Institute Osaka Japan; ^2^ Department of Genetic Oncology Division of Hereditary Tumors Osaka International Cancer Institute Osaka Japan; ^3^ Endoscopy Center Osaka International Cancer Institute Osaka Japan

**Keywords:** adverse events, colonic neoplasms, colonic polyps, colonoscopic surgery, colonoscopy

## Abstract

Underwater endoscopic mucosal resection (UEMR) is a newly developed technique for the removal of colorectal, duodenal, esophageal, gastric, ampullary, and small intestinal lesions. We performed a PubMed literature search for articles reporting UEMR outcomes for colorectal polyps. Four randomized controlled trials, nine non‐randomized prospective trials, 16 retrospective studies, and 27 case reports were selected for assessment of the efficacy and safety of UEMR. We summarized the therapeutic outcomes of UEMR in each category according to the lesion characteristics [small size (<10 mm), intermediate size (10–19 mm), large size (≥20 mm), recurrent lesion, and rectal neuroendocrine tumor], and calculated the incidence of adverse events among the included articles. As the treatment outcomes for small polyps appeared similar between UEMR and conventional endoscopic mucosal resection (CEMR), UEMR can be a standard procedure for small colorectal polyps suspicious for high‐grade dysplasia to avoid incomplete removal of occult invasive cancer by cold snare polypectomy. As UEMR showed satisfactory outcomes for intermediate‐size lesions and recurrent lesions after endoscopic resection, UEMR can be a standard procedure for these lesions. Regarding large lesions and rectal neuroendocrine tumors, comparisons of UEMR with current standard methods for them were lacking, and further investigations are warranted. Adverse events appeared comparable or less frequent for UEMR compared with CEMR but still existed. Therefore, careful implementation of this new technique in clinical practice is important for its widespread use.

## BACKGROUND

Endoscopic removal of colorectal lesions started with the simple snarectomy reported in 1971.^1^ Conventional endoscopic mucosal resection (CEMR) after submucosal injection and other modified techniques were subsequently developed, and further developments seemed unlikely. Nevertheless, cold snare polypectomy (CSP), a polypectomy procedure without electrocautery, has had a large impact on clinical practice because of its efficacy^2^ and safety,^3^ leading to the so‐called “Cold Revolution”.^4^ Underwater endoscopic mucosal resection (UEMR), initially reported in 2012,^5^ was also expected to have a great impact. UEMR was reported to be effective in the treatment of duodenal polyps.^6^, ^7^ Furthermore, UEMR could be integrated with endoscopic interventions for esophageal,^8^ gastric,^9^ ampullary,^10^ and small intestinal^11^ lesions.

European Society of Gastrointestinal Endoscopy clinical guidelines for colorectal polypectomy and EMR^12^ and the recommendations for endoscopic removal of colorectal lesions from the US Multi‐Society Task Force^13^ have mentioned UEMR briefly, but they have not indicated the degree of recommendation since it did not have enough evidence. In this article, we reviewed the reported articles and summarized the effectiveness and safety of UEMR for colorectal lesions. As the indications and outcomes of UEMR varied among the studies, we categorized the articles and summarized the findings according to the characteristics of the removed lesions.

## LITERATURE SEARCH AND ARTICLE SELECTION

We performed a literature search of PubMed for all studies reporting the complete resection rate (CRR), *en bloc* resection rate (EBR), or adverse events of UEMR for colorectal polyps. We used the following keywords for the PubMed search: (“underwater”[All Fields] AND (“endoscopic mucosal resection”[MeSH Terms] OR (“endoscopic”[All Fields] AND “mucosal”[All Fields] AND “resection”[All Fields]) OR “endoscopic mucosal resection”[All Fields])) OR (“underwater”[All Fields] AND (“empir musicol rev”[Journal] OR “emr”[All Fields])). The language for the search was restricted to English. The date of the last search was 20 September 2021.

One researcher (Yoji Takeuchi) reviewed all identified records for inclusion using the title and abstract. The inclusion criteria were English full‐text articles reporting CRR, incomplete resection rate (IRR), EBR, or adverse events of UEMR for colorectal lesions in prospective studies, retrospective studies, and case reports.

**FIGURE 1:**
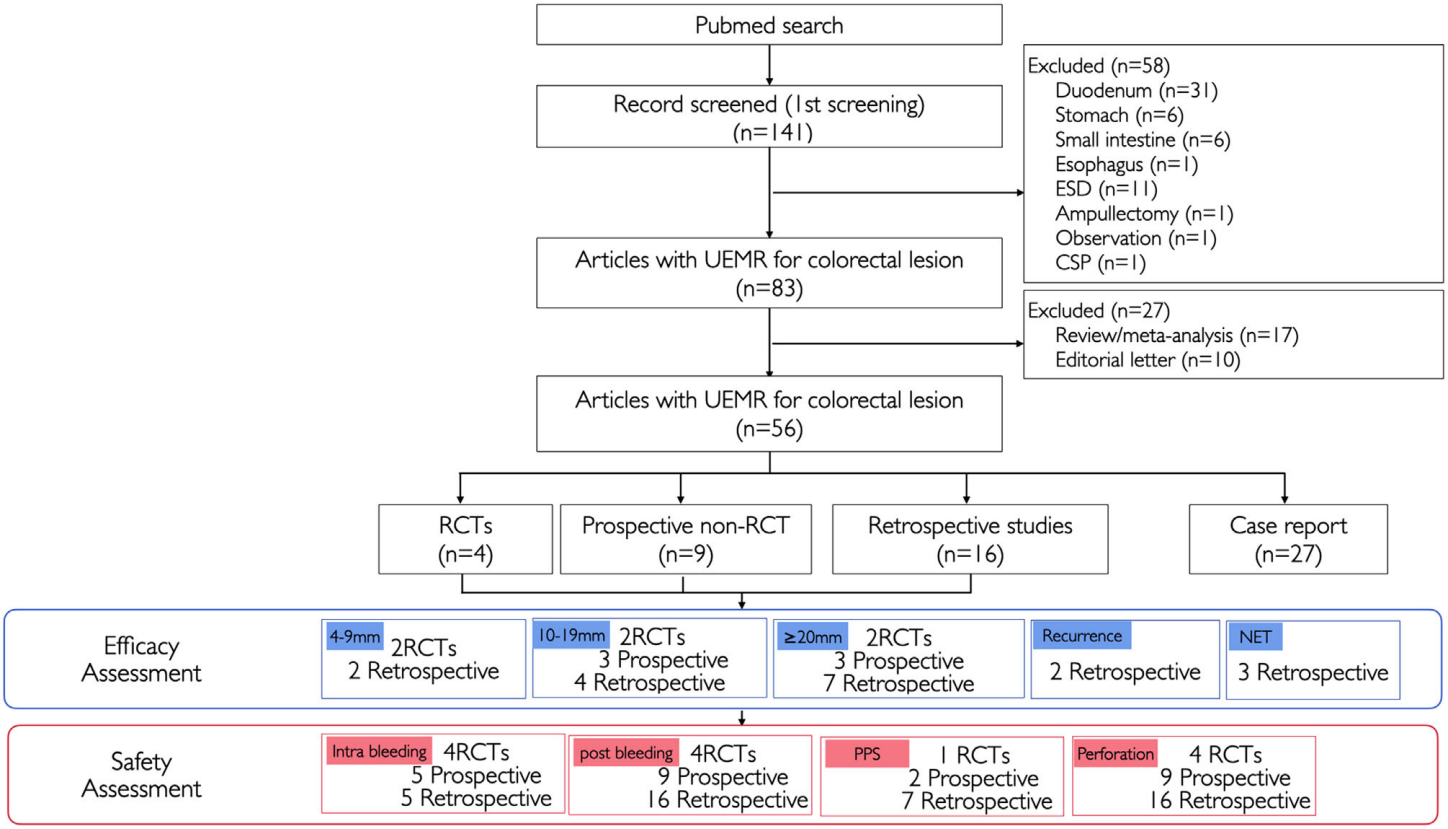
Flow chart of the study selection process. RCT, randomized controlled trial; CSP, cold snare polypectomy; Intra bleeding, intraprocedural bleeding; Post bleeding, postprocedural bleeding

We identified a total of 141 records through the database search (Figure 1). There was no removal of duplicates, and all 141 records were assessed for the title and abstract. We excluded 44 articles, not on colorectal lesions (31 duodenum, six stomach, six small intestine, and one esophagus), 14 other procedures (11 endoscopic submucosal dissection [ESD], one CSP, one ampullectomy, and one observation), 17 reviews involving meta‐analyses, and 10 editorials or letters. Finally, we assessed 56 full‐text articles comprising four randomized controlled trials (RCTs), nine non‐randomized prospective trials, 16 retrospective studies, and 27 case reports for the efficacy and safety of UEMR for colorectal lesions.

## DEFINITION OF TERMINOLOGY

CEMR in all selected articles is defined as filling the colonic lumen with air or CO_2_ with submucosal injection of fluid underneath the polyp followed by snare resection of the lesion. UEMR included snare resection of the lesion without submucosal injection after suctioning the gas in the colonic lumen and infusing sterile water or natural saline. Complete resection was defined as EBR with a negative pathological margin. *En bloc* resection was defined as a single‐piece resection without remnant polyps on endoscopic view. Adverse events included perforation or hemorrhage requiring blood transfusion, endoscopic treatment, and/or surgery. Although the definition of bleeding varied from article to article, intraprocedural bleeding was defined as bleeding that required hemostatic treatment during the procedure, and postprocedural bleeding was defined as any bleeding that occurred after the procedure.

We summarized the therapeutic outcomes of UEMR in each category according to the lesion characteristics. The characteristics were categorized as small size (< 10 mm), intermediate size (10−19 mm), large size (≥20 mm), recurrent lesion, and rectal neuroendocrine tumor (NET). Because the inclusion criteria in each study were different, we adopted articles that clearly indicated the outcomes of UEMR in each category. Regarding adverse events, we investigate the incidence of intraprocedural bleeding, postprocedural bleeding, post‐polypectomy coagulation syndrome, and perforation. As some adverse events were not clearly described, we selected articles that clearly described the incidence or number of each adverse event. We calculated the incidence of each adverse event by dividing the total number of reported events by the total number of included cases in the selected articles for each category.

## OUTCOMES OF UEMR FOR SMALL‐SIZE (<10 mm) COLORECTAL POLYPS

Table 1 shows literature reports assessed for the efficacy of underwater endoscopic mucosal resection for small (<10 mm) colorectal polyps. One multicenter RCT compared the outcomes of UEMR and CEMR for 4–9‐mm colorectal polyps.^14^ Another single‐center RCT comparing UEMR and CEMR for colorectal polyps larger than 6 mm assessed the outcomes for 6–9‐mm lesions in a subgroup analysis.^15^ Two retrospective studies enrolled patients with colorectal polyps of any size and showed the outcomes for 6–9‐mm lesions in a subgroup analysis.^16^, ^17^

**TABLE 1:** Literature reports assessed for the efficacy of underwater endoscopic mucosal resection for small (<10 mm) colorectal polyps

	**First author**	**Study design**	**Procedure**	**Included lesions**	**No. of institutes**	**Country**	**No. of polyps (UEMR)**	CRR (UEMR)	CRR (CEMR)	EBR (UEMR)	EBR (CEMR)
12	Zhang	Multicenter RCT	UEMR/CEMR	4–9 mm	3	China	66	83.1%	87.3%	94.4%	91.5%
13	Yen	Single‐center RCT	UEMR/CEMR	6–9 mm,>10 mm	1	USA	180	–	–	97.2%	99.4%
14	Cadoni	Retrospective	UEMR/CEMR	Any size	2	Italy	27	100%	100%	100%	100%
19	Kawamura	Retrospective	UEMR	Any size	1	Japan	8	88%	–	100%	–

Abbreviations: CEMR, conventional endoscopic mucosal resection; CRR, complete resection rate; EBR, en bloc resection rate; RCT, randomized controlled trial; UEMR, underwater endoscopic mucosal resection.

The multicenter RCT showed non‐inferior CRR and EBR for UEMR (83% and 94%) compared with CEMR (87% and 92%).^14^ In the RCT, two postprocedural bleeding events were observed after CEMR, compared with none after UEMR. Therefore, the authors concluded that UEMR is safer than and as effective as CEMR and can be recommended as an alternative approach to excision for small and non‐pedunculated colorectal adenomatous polyps. The single‐center RCT showed non‐significant differences in IRR indicated by additional biopsy and EBR between UEMR (1.7% and 97%) and CEMR (2.4% and 99%) for 6–9‐mm colorectal polyps.^15^ It also found a significantly shorter procedure time for UEMR (1.3 min) compared with CEMR (1.1 min). Of the two retrospective studies, one showed similar CRR and EBR for UEMR (100% and 98%) and CEMR (100% and 100%),^16^ while the other showed satisfactory high CRR and EBR (88% and 100%) for UEMR.^17^ Therefore, the treatment outcomes for small polyps appear similar between UEMR and CEMR. Besides, UEMR can decrease the cost for the endoscopic needle and may reduce the procedure time. Therefore, UEMR has advantages for cost‐effectiveness compared with CEMR.

**FIGURE 2:**
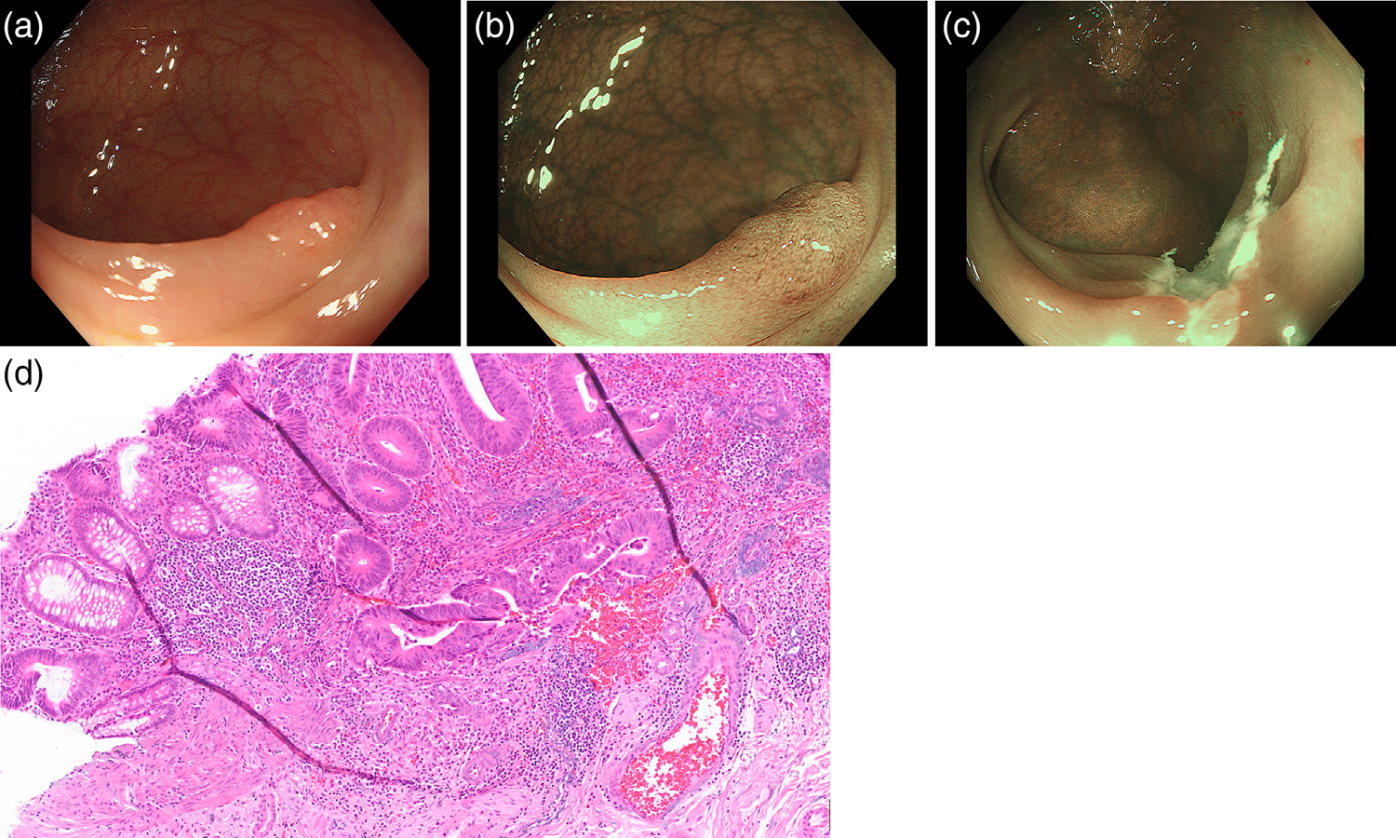
A small sessile lesion in the transverse colon removed by underwater endoscopic mucosal resection (UEMR). (a) White‐light image of an 8‐mm sessile lesion in the transverse colon. The surface of the lesion appears slightly irregular. (b) Magnified narrow‐band image of the lesion in the dual‐focus mode. The microvessels appear irregular, indicating high‐grade dysplasia (JNET Type 2B). (c) Mucosal defect after UEMR. (d) Histopathological findings of the resected specimen, indicating minute invasion (200 μm)

CSP is recommended as the standard procedure for small polyps.^12^, ^13^ However, it should not be adopted for endoscopically diagnosed high‐grade adenoma because its superficial incision can lead to a risk for incomplete incision of invasive cancer.^18^ Meanwhile, the dissection depth of UEMR is comparable to that of CEMR.^19^ Therefore, UEMR can be a standard procedure for small colorectal polyps suspicious for high‐grade dysplasia to avoid incomplete removal of occult invasive cancer (Figure 2, Video 1).

## OUTCOMES OF UEMR FOR INTERMEDIATE‐SIZE (10–19 mm) COLORECTAL POLYPS

Table 2 shows literature reports assessed for the efficacy of underwater endoscopic mucosal resection for intermediate‐size (10–19 mm) colorectal polyps. One multicenter RCT compared UEMR and CEMR for intermediate‐size colorectal polyps.^20^ Another single‐center RCT comparing UEMR and CEMR for colorectal polyps larger than 6 mm assessed the outcomes for intermediate‐size lesions in a subgroup analysis.^15^ Two prospective observational single‐arm studies including lesions larger than 10 mm^21^, ^22^ and five retrospective studies^16^, ^17^, ^23^, ^24^, ^25^ showed the outcomes of UEMR for intermediate‐size polyps in a subgroup analysis.

**TABLE 2:** Literature reports assessed for the efficacy of underwater endoscopic mucosal resection for intermediate‐size (10–19 mm) colorectal polyps

	**First author**	**Study design**	**Procedure**	**Included lesions**	**No. of institutes**	**Country**	**No. of polyps (UEMR)**	**CRR (UEMR)**	**CRR (CEMR)**	**EBR (UEMR)**	**EBR (CEMR)**
19	Yamashina	Multicenter RCT	UEMR/CEMR	10–19 mm	5	Japan	108	69%	50%	89%	75%
13	Yen	Single‐center RCT	UEMR/CEMR	6–9 mm,>10 mm	1	USA	52	–	–	84.6%	73.5%
20	Siau	Prospective Multicenter	UEMR	>10 mm	2	UK	–	–	–	82.9%	–
21	Amato	Prospective	UEMR	>10 mm	1	Italy	7	100%	–	100%	–
14	Cadoni	Retrospective	UEMR/CEMR	Any size	2	Italy	63	77.8%	79.3%	81%	79.3%
15	Kawamura	Retrospective	UEMR	Any size	1	Japan	34	63%	–	94%	–
22	Chien	Retrospective	UEMR/CEMR	≥10 mm	1	Taiwan	98			97.3%	96.0%
23	Chaves	Retrospective	UEMR	≥10 mm	1	Brazil	6	83.3%	–	–	–
24	Schenck	Retrospective	UEMR/CEMR	≥15 mm	1	USA	19	–	–	–	–

Abbreviations: CEMR, conventional endoscopic mucosal resection; CRR, complete resection rate; EBR, en bloc resection rate; RCT, randomized controlled trial; UEMR, underwater endoscopic mucosal resection.

The multicenter RCT showed superior CRR and EBR for UEMR (69% and 89%) compared with CEMR (50% and 76%) as well as comparable procedure times (165 vs. 175 s) and adverse events (2.8% vs. 2.0%).^20^ The single‐center RCT showed non‐significant differences in IRR indicated by additional biopsy and EBR between UEMR (1.9% and 85%) and CEMR (0% and 74%).^15^ It also found a significantly shorter procedure time for UEMR (2.9 min) compared with CEMR (5.6 min). Both of the two prospective single‐arm studies showed satisfactory high EBR (83% and 100%) for intermediate‐size colorectal polyps.^21^, ^22^ Three of the five retrospective studies compared UEMR and CEMR.^16^, ^23^, ^25^ While all three studies showed comparable CRR and/or EBR or local recurrence rate between UEMR and CEMR, two found a significantly shorter procedure time for UEMR compared with CEMR, while the third did not indicate the procedure times. The other two retrospective studies were both single‐arm studies, and also showed satisfactory high CRR (83% and 63%) and EBR (83% and 94%) for UEMR.^17^, ^24^

**FIGURE 3:**
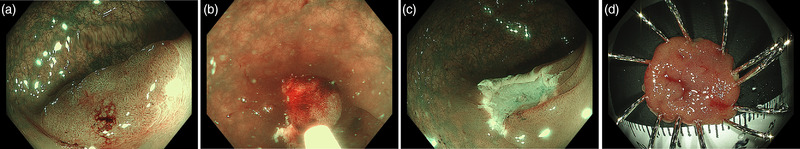
An intermediate‐size sessile lesion in the sigmoid colon removed by underwater endoscopic mucosal resection (UEMR). (a) Narrow‐band image of a 20‐mm non‐granular‐type laterally‐spreading tumor in the sigmoid colon. (b) Snaring without submucosal injection underwater. (c) Mucosal defect after UEMR. (D) Resected specimen. Pathological findings indicated high‐grade adenoma

While the two RCTs showed split results on EBR for UEMR compared with CEMR, one of them was a subgroup analysis involving a small number of intermediate‐size colorectal lesions without a sample size calculation. Thus, the subgroup analysis may have a type II error (false‐negative results). At least, the EBR for intermediate‐size colorectal lesions with UEMR appeared non‐inferior to CEMR, and the pivotal results in several prospective and retrospective studies indicated a shorter procedure time for UEMR compared with CEMR. Therefore, UEMR can be one of the standard procedures for intermediate‐size colorectal polyps (Figure 3, Video 2).

## OUTCOMES OF UEMR FOR LARGE‐SIZE (≥20 mm) COLORECTAL POLYPS

Table 3 shows literature reports assessed for the efficacy of underwater endoscopic mucosal resection for large (>20 mm) colorectal polyps. One single‐center RCT compared UEMR and CEMR for large colorectal polyps.^26^ Another single‐center RCT comparing UEMR and CEMR for colorectal polyps larger than 6 mm assessed the outcomes of large lesions in a subgroup analysis.^15^ One prospective study^5^ and two retrospective studies investigated the outcomes of UEMR for lesions larger than 20 mm.^27^, ^28^ Two prospective studies including lesions larger than 5 and 10 mm,^22^, ^29^ two retrospective studies including lesions larger than 10 mm,^23^, ^24^ two retrospective studies including lesions larger than 15 mm,^25^, ^30^ and one retrospective study including polyps of any size^16^ showed the outcomes of UEMR for large lesions in a subgroup analysis.

**TABLE 3:** Literature reports assessed for the efficacy of underwater endoscopic mucosal resection for large (>20 mm) colorectal polyps

	**First author**	**Study design**	**Procedure**	**Included lesions**	**No. of institutes**	**Country**	**No. of polyps (UEMR)**	**CRR (UEMR)**	**CRR (CEMR)**	**EBR (UEMR)**	**EBR (CEMR)**
25	Nagl	Single‐center RCT	UEMR/CEMR	20–40 mm	1	Germany	73	32.1%	15.8%	33.3%	18.8%
13	Yen	Single‐center RCT	UEMR/CEMR	6–9 mm,>10 mm	1	USA	52	–	–	25.0%	43.8%
26	Binmoeller	Single‐center prospective	UEMR	>20 mm	1	USA	62	–	–	–	–
27	Inoue	Retrospective	UEMR/ESD	20–30 mm	1	Japan	125	36%	–	61%	–
28	Barclay	Retrospective	UEMR/CEMR	≥20 mm	1	USA	264	–	–	–	–
29	Nogueira	Prospective	UEMR	>5 mm	1	Brazil	8	–	–	23.5%	–
30	Uedo	Retrospective	UEMR	≥15 mm	1	Sweden	11	64%	–	55%	–
21	Amato A	Prospective	UEMR	>10 mm	1	Italy	18	–	–	67%	–
22	Chien HC	Retrospective	UEMR/CEMR	≥10 mm	1	Taiwan	42	–	–	69.1%	52.4%
23	Chaves DM	Retrospective	UEMR	≥10 mm	1	Brazil	10	40%	–	–	–
24	Schenck RJ	Retrospective	UEMR/CEMR	≥15 mm	1	USA	29	–	–	–	–
14	Cadoni S	Retrospective	UEMR/CEMR	Any size	2	Italy	18	38.9%	26.3%	38.9%	26.3%

Abbreviations: CEMR, conventional endoscopic mucosal resection; CRR, complete resection rate; EBR, en bloc resection rate; RCT, randomized controlled trial; UEMR, underwater endoscopic mucosal resection.

The single‐center RCT comparing UEMR and CEMR for 20–40‐mm lesions directly showed superior CRR and EBR for UEMR (32% and 33%) compared with CEMR (16% and 18%) as well as a significantly shorter procedure time (8 vs. 14 min) and fewer pieces for piecemeal resection (two pieces: 46% for UEMR vs. 18% for CEMR).^26^ However, the overall recurrence rates did not differ between the two groups. The other single‐center RCT showed non‐significant differences in IRR indicated by additional biopsy and EBR between UEMR (6.3% and 25%) and CEMR (0% and 44%).^15^ It also found a significantly shorter procedure time for UEMR (7.3 min) compared with CEMR (9.5 min). All three prospective studies were observational single‐arm studies without a control arm. Although the EBRs in these prospective studies (24%–66%) were not satisfactory,^22^, ^29^ the acceptable adverse events, procedure times, and low recurrence rates with UEMR allowed the conclusion that the procedure was feasible.^5^ Three of the seven retrospective studies compared UEMR and CEMR.^16^, ^23^, ^25^ While two of these studies showed better, albeit statistically insignificant, CRR and/or EBR for UEMR compared with CEMR,^16^, ^23^ one showed a significantly smaller local recurrence rate for UEMR compared with CEMR. The other four retrospective single‐arm studies showed satisfactory EBR (30%–60%) and low local recurrence rate (0%–5.7%) for UEMR.^24^, ^27^, ^28^, ^30^

**FIGURE 4:**
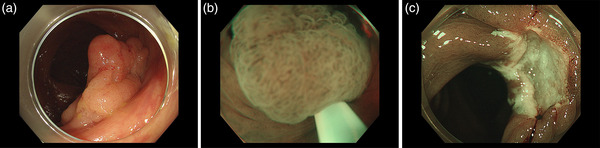
A large sessile lesion in the ascending colon removed by underwater endoscopic mucosal resection (UEMR). (a) White‐light image of a 30 mm granular‐type laterally‐spreading tumor in the ascending colon. (b) Snaring without submucosal injection underwater. (c) Mucosal defect after UEMR

While the two RCTs showed split results on EBR for UEMR compared with CEMR, one of them was a subgroup analysis involving a small number of large colorectal lesions without a sample size calculation. The results of this subgroup analysis may have a Type II error (false‐negative results). The pivotal results in several studies may support the superiority of UEMR compared with CEMR in terms of CRR, EBR, and shorter procedure time. However, regarding large colorectal lesions, ESD is the most promising procedure in terms of CRR, EBR, and local recurrence rate. Only one retrospective study with propensity score matching showed comparability of UEMR with ESD for 20–30‐mm colorectal lesions.^28^ Therefore, future prospective studies are warranted to determine the relevance of UEMR for large colorectal lesions (Figure 4, Video 3).

## OUTCOMES OF UEMR FOR RECURRENT LESIONS AFTER ENDOSCOPIC RESECTION

Table 4 shows literature reports assessed for the efficacy of underwater endoscopic mucosal resection for recurrent colorectal polyps. Only two retrospective studies investigated the efficacy of UEMR for recurrent lesions after endoscopic resection.^31^, ^32^ One retrospective analysis compared 36 UEMR and 44 CEMR procedures.^32^ It showed that UEMR had significantly better EBR (47% vs. 16%) and endoscopic CRR (89% vs. 32%) than CEMR. The recurrence rate after the intervention was significantly lower for UEMR (11%) compared with CEMR (66%). Therefore, UEMR would be advantageous for recurrent colorectal lesions after endoscopic resection compared with CEMR.

**TABLE 4:** Literature reports assessed for the efficacy of underwater endoscopic mucosal resection for recurrent colorectal polyps

	**First author**	**Study design**	**Procedure**	**Included lesions**	**No. of institutes**	**Country**	**No. of polyps (UEMR)**	**CRR (UEMR)**	**CRR (CEMR)**	**EBR (UEMR)**	**EBR (CEMR)**
31	Ohmori	Retrospective	UEMR/ESD	Recurrent	1	Japan	30	73%	–	41%	–
32	Kim	Retrospective	UEMR/CEMR	Recurrent	1	USA	36	–	–	47.2%	15.9%

Abbreviations: CEMR, conventional endoscopic mucosal resection; CRR, complete resection rate; EBR, en bloc resection rate; RCT, randomized controlled trial; UEMR, underwater endoscopic mucosal resection.

**FIGURE 5:**
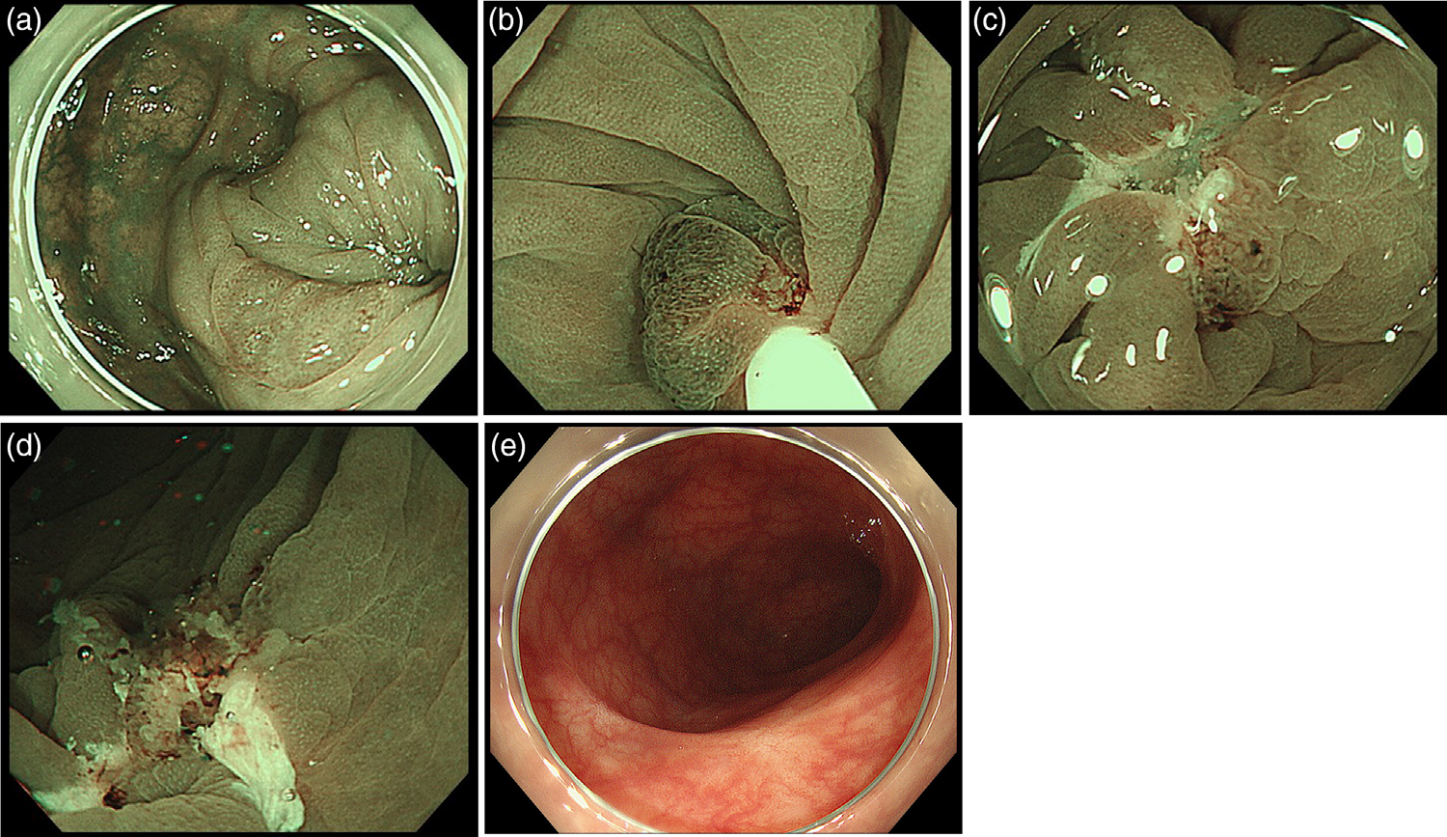
A recurrent polyp after polypectomy in the sigmoid colon removed with piecemeal underwater endoscopic mucosal resection (UEMR). (a) A narrow‐band image of an 8 mm, recurrent polyp at the sigmoid colon, accompanied with scar after previous endoscopic polypectomy at the anal side of the lesion. (b) Snaring without submucosal injection underwater. (c) Mucosal defect after UEMR with a small remnant. (d) Mucosal defect after additional UEMR. (e) Endoscopic image of the scar after UEMR at surveillance colonoscopy 1 year later

Because ESD can remove recurrent lesions after a previous attempt for endoscopic mucosal resection, UEMR and ESD were compared in another retrospective study with propensity score matching.^31^ Although ESD had significantly better EBR and CRR than UEMR (100% vs. 73% and 81% vs. 41%, respectively), two delayed perforations were seen in 10% of ESD cases and UEMR had a significantly shorter procedure time and hospitalization period compared with ESD. Meanwhile, local recurrence was not seen in either group. Because the lesion size in the propensity score matching was small (median: 10 mm), we consider that the results can be adopted for non‐large (less than 15−20 mm) colorectal lesions (Figure 5, Video 4).

## OUTCOMES OF UEMR FOR RECTAL NET

Table 5 shows literature reports assessed for the efficacy of underwater endoscopic mucosal resection for rectal neuroendocrine tumors. UEMR may have advantages for not only colorectal epithelial lesions but also submucosal tumors, such as NET in the rectum. Three retrospective studies investigated the efficacy of UEMR for rectal NET (Table 5).^33^, ^34^, ^35^ Park et al.^34^ compared UEMR and ESD for rectal NET. They enrolled 115 patients, of whom 36 underwent UEMR and 79 underwent ESD. CRR for both procedures was 86%. UEMR had a significantly shorter procedure time and fewer adverse events. Coutinho et al.^33^ investigated the outcomes of UEMR for rectal NET in 11 patients. Although EBR was 100%, two patients had deep margin involvement. Thus, CRR was 81%. Yamashina et al.^35^ investigated six consecutive patients and reported EBR of 100% and CRR of 83%.

**TABLE 5:** Literature reports assessed for the efficacy of underwater endoscopic mucosal resection for rectal neuroendocrine tumors

	**First author**	**Study design**	**Procedure**	**Included lesions**	**No. of institutes**	**Country**	**No. of polyps (UEMR)**	**CRR (UEMR)**	**CRR (CEMR)**	**EBR (UEMR)**	**EBR (CEMR)**
33	Coutinho	Retrospective	UEMR	NET	1	Brazil	11	81%	–	100%	–
34	Kim	Retrospective	UEMR/ESD	NET	1	USA	36	86.1%	86.1%‐	–	–
35	Yamashina	Retrospective	UEMR	NET	1	Japan	6	83	–	100%	–

Abbreviations: CEMR, conventional endoscopic mucosal resection; CRR, complete resection rate; EBR, en bloc resection rate; NET, neuroendocrine tumor; RCT, randomized controlled trial; UEMR, underwater endoscopic mucosal resection.

For rectal NET treated by UEMR, EBR was 100% and CRR was 81%–86% in the above reports. These results appear comparable to those for ESD, and thus UEMR can be an alternative to ESD. However, endoscopic submucosal resection with a ligation device (ESMR‐L) allowed incision beneath the ligated band under the submucosal tumor and provided a high CRR (99%) in a retrospective study.^36^ Therefore, the reliability of ESMR‐L for CRR in rectal NET is theoretically high compared with that of UEMR, even though UEMR has an advantage for reduced cost for the device compared with ESMR‐L.

## UEMR IN CASE REPORTS UNDER SPECIAL SITUATIONS

UEMR was described as being useful in case reports under special situations. Two case reports showed successful resection with UEMR for complete removal of adenoma with extension into an appendiceal orifice.^37^, ^38^ Furthermore, a prospective study on clinical outcomes of UEMR for lesions involving an appendiceal orifice found that UEMR was successful in 89% (24/27) with the post‐polypectomy syndrome in 7% and local recurrence rate of 10%.^39^ Three case reports indicated complete removal of adenoma extending into a colonic diverticulum or surrounded by a diverticulum using UEMR.^40^, ^41^, ^42^ Appendiceal orifice and diverticulum are similar conditions involving deep depression of the colonic wall, and these case reports may indicate the efficacy of a “floating” effect in UEMR.

Six case reports showed successful UEMR for lesions in the lower rectum and anal canal.^43^, ^44^, ^45^, ^46^, ^47^, ^48^ The lower rectum and anal canal are narrow, and the working space for endoscopic procedures is limited, especially when submucosal injection is performed. UEMR can be performed without submucosal injection and would not interfere with snaring in the narrow lumen. One retrospective case series^49^ and one case report for a lesion in the ileocecal valve^50^ also showed the significance of snaring without submucosal injection. If the submucosal injection was carried out, a lesion in such a complicated location would be slippery for snaring. These case reports demonstrate the effectiveness of snaring without submucosal injection in UEMR.

Four case reports described UEMR for lesions accompanied by a scar.^51^, ^52^, ^53^, ^54^ Although this advantage of UEMR was already described in retrospective analyses, the technique and efficacy are worthy of publication as case reports for provision of information because these lesions are difficult to remove using conventional procedures like CEMR or ESD. Two other case reports described UEMR in patients with ulcerative colitis.^55^, ^56^ The persistent inflammation in ulcerative colitis causes submucosal fibrosis that makes endoscopic removal difficult by CEMR or ESD. Therefore, these case reports demonstrate one of the greatest advantages of UEMR, namely its efficacy for the removal of lesions with fibrosis.

One case report described UEMR for rectal adenoma in the non‐distensible rectum arising from severe fecal incontinence,^57^ and indicated that UEMR can even be successful in situations where the performance of CEMR and ESD is difficult. However, some endoscopists remained skeptical about UEMR as a special procedure and investigated techniques to decrease the difficulty of UEMR in case reports that utilized peristalsis,^58^ a snaring technique,^59^ and a special device.^60^ Meanwhile, Wang et al.^61^ conducted a prospective study and showed that UEMR can be easily learned by endoscopists who are already skilled in CEMR. Therefore, more evidence and information about the efficacy and safety of UEMR are mandatory to make UEMR a common procedure.

## SAFETY (ADVERSE EVENTS) OF UEMR

Four RCTs, five prospective studies, and five retrospective studies reported the incidence of intraprocedural bleeding. A total of 1222 polyps were included and 63 postprocedural bleeding events were reported. Thus, the incidence of intraprocedural bleeding was estimated at 5.2% (95% confidence interval, 4.0%–6.6%). Because the definition of intraprocedural bleeding and the included lesions differed among the studies, the actual incidence of intraprocedural bleeding ranged from 0% to 14.5%, but all intraprocedural bleeding events were controlled by endoscopic intervention. The method for endoscopic hemostasis of intraprocedural bleeding after UEMR has not been unified, but any hemostasis, such as endo‐clip or electrocautery, can be adopted. Therefore, we should keep in mind that intraprocedural bleeding can occur during UEMR and we should prepare a preferred device for hemostasis before UEMR.

All of the included RCTs, prospective studies, and retrospective studies reported the incidence of postprocedural bleeding. A total of 2101 polyps were included and 29 postprocedural bleeding events were reported. Thus, the incidence of intraprocedural bleeding is estimated at 1.4% (95% confidence interval, 0.9%–2.0%). The incidence differed according to the lesion characteristics, such as size or morphology, but at least the incidence of postprocedural bleeding for UEMR appeared comparable to that for CEMR in all RCTs. Therefore, the risk of postprocedural bleeding should be informed as well as the risk of CEMR before the performance of UEMR.

Regarding post‐polypectomy coagulation syndrome, only one RCT, two prospective studies, and seven retrospective studies reported its incidence. A total of 860 polyps were included and only six post‐polypectomy coagulation syndrome events were reported. Thus, the incidence of post‐polypectomy coagulation syndrome was estimated at 0.7% (95% confidence interval, 0.2%–1.5%). Because the incidence of the post‐polypectomy syndrome after CEMR varied from 0.2% to 7.6%,^62^ the incidence of the post‐polypectomy syndrome after UEMR appeared acceptable, considering that the studies on UEMR included many large lesions. UEMR can theoretically be expected to decrease the incidence of the post‐polypectomy syndrome because of its heat‐sink effect.

Because perforation is the main issue for colorectal polypectomy, all of the included RCTs, prospective studies, and retrospective studies reported the incidence of perforation. A total of 2101 polyps were included and only six perforation events were reported. Thus, the incidence of perforation was estimated at 0.3% (95% confidence interval, 0.1%–0.6%). The occurrence of perforation after UEMR was generally recognized to be low and may thus be limited to case reports because of its rarity.^63^, ^64^ Theoretically, however, there are no EMR procedures without any risk of perforation. In our personal experience, there is a higher risk of perforation when larger lesions are treated by UEMR. Therefore, we would like to recommend having endo‐clips on standby for wound closure when performing UEMR for large colorectal polyps.

## CONCLUSIONS

UEMR can be a standard procedure for small polyps suspicious for high‐grade dysplasia, intermediate‐size polyps, and recurrent lesions in the colorectum. Further studies are warranted to show the efficacy of UEMR for large lesions compared with ESD. Theoretically, we need to be reluctant for UEMR to become a standard procedure for rectal NET. Adverse events appear comparable or less frequent for UEMR compared with CEMR but still exist. Therefore, careful implementation of this new technique in clinical practice is important for its widespread use.

## CONFLICT OF INTEREST

Yoji Takeuchi has received honoraria for lectures from Olympus, Boston Scientific Japan, Daiichi‐Sankyo, Miyarisan Pharmaceutical, Asuka Pharmaceutical, AstraZeneca, EA Pharma, Zeria Pharmaceutical, Fujifilm, Kaneka Medix, Kyorin Pharmaceutical, and The Japan Gastroenterological Endoscopy Society. Satoki Shichijo has received honoraria for lectures from Olympus, Boston Scientific Japan, Daiichi Sankyo, EA Pharma, Zeria Pharmaceutical, The Japanese Society of Gastroenterology, and The Japan Gastroenterological Endoscopy Society. Noriya Uedo has received personal fees from Olympus, Fujifilm, Boston Scientific Japan, 3‐D Matrix Ltd., Daiichi Sankyo, Takeda Pharmaceutical, EA Pharma, Otsuka Pharmaceutical, AstraZeneca, Top Cooperation, and Miyano Medical Instruments. Ryu Ishihara has received personal fees from EA Pharma, AstraZeneca, Ono Pharmaceutical, MSD, Olympus, Daiichi Sankyo, and Fujifilm. These organizations had no role in the design, practice, or analysis in this manuscript. Yoji Takeuchi is an associate editor of DEN Open.

## FUNDING INFORMATION

The manuscript was supported by a grant from the Practical Research for Innovative Cancer Control (JP 21ck0106556) from the Japan Agency for Medical Research and Development (AMED). The funding source had no role in the design, practice, or analysis of the study.

## Supporting information




**Video 1**. Underwater endoscopic mucosal resection for a small sessile lesion in the transverse colon, which was suspicious for high‐grade dysplasia.

**Video 2**. Underwater endoscopic mucosal resection for an intermediate size sessile lesion in the sigmoid colon.

**Video 3**. Underwater endoscopic mucosal resection for a large sessile lesion in the ascending colon.

**Video 4**. Underwater endoscopic mucosal resection for a recurrent polyp after polypectomy in the sigmoid colon.
